# Associations between dynamic-contrast enhanced MRI and tumor infiltrating lymphocytes and tumor-stroma ratio in head and neck squamous cell cancer

**DOI:** 10.1186/s40644-021-00429-z

**Published:** 2021-11-20

**Authors:** Hans-Jonas Meyer, Anne Kathrin Höhn, Alexey Surov

**Affiliations:** 1 Department of Diagnostic and Interventional Radiology, University of Leipzig, Leipzig, Germany; 2 Department of Pathology, University of Leipzig, Leipzig, Germany; 3 Department of Radiology and Nuclear Medicine, University of Magdeburg, Magdeburg, Germany

**Keywords:** Head and neck squamous cell cancer, Dynamic contrast enhanced-MRI, Tumor infiltrating lymphocyte, Histogram analysis

## Abstract

**Objectives:**

The present study used dynamic-contrast enhanced MRI (DCE-MRI) to elucidate possible associations with tumor-infiltrating lymphocytes (TIL), stroma ratio and vimentin expression in head and neck squamous cell cancer (HNSCC).

**Methods:**

Overall, 26 patients with primary HNSCC of different localizations were involved in the study. DCE-MRI was obtained on a 3 T MRI and analyzed with a whole lesion measurement using a histogram approach. TIL- and vimentin-expression was calculated on bioptic samples before any form of treatment. P16 staining was used to define HPV-status.

**Results:**

Tumor-stroma ratio correlated with entropy derived from K^trans^ (*r* = − 0.52, *p* = 0.0071) and with kurtosis derived from V_e_ (*r* = − 0.53, *p* = 0.0058).

Several V_e_ derived parameters correlated with expression of TIL within the stroma compartment. TIL within the tumor compartment correlated with entropy derived from K^trans^ (*r* = 0.39, *p* = 0.047), p90 derived from V_e_ (*r* = 0.41, *p* = 0.036) and skewness derived from V_e_ (*r* = 0.41, *p* = 0.037). Furthermore, these associations were different between HPV positive and negative tumors.

**Conclusions:**

DCE-MRI might be able to reflect tumor compartments and TIL expression in HNSCC. The most promising parameters were values derived from K^trans^ and V_e_.

## Introduction

Head and neck squamous cell cancer (HNSCC) is the sixth most common malignancy worldwide with a rising incidence [1, 2]. Approximately 600,000 new cases of HNSCC patients and over 33,000 HNSCC related deaths per year [2]. Modern functional imaging modalities, comprising diffusion-weighted imaging (DWI) and Dynamic-contrast enhanced magnetic resonance imaging (DCE MRI) give further insight into tumor microstructure in a non-invasive manner [3–8]. Modern MRI can predict several histological features of tumors, treatment success and provide prognostic biomarkers [3–8]. DCE-MRI quantifies the time dependent influx of contrast media into tissues [9, 10]. In most studies, the investigated parameters are volume transfer constant or K^trans^, rate constant or K_ep_ and volume fraction of the extravascular extracellular space or V_e_ [8, 9].

According to the literature, these parameters can reflect several underlying histopathological features [4]. For example, V_e_ is more related to cell density, whereas K^trans^ can reflect proliferation potential and microvessel density [4, 10, 11].

There is growing interest on the field regarding tumor-infiltrating lymphocytes (TIL) around oncology [12, 13]. The complex tumor microenvironment is of special interest as the communication between tumor and immune cells modulates tumor progress and treatment response [14]. Importantly, TILs are an independent prognostic factor in patients with HNSCC [12]. Another clinical relevant histopathological feature is the.

tumor-stroma ratio [15–17]. A low tumor-stroma ratio implies a relatively high quantity.

of stroma, which has been shown to be an adverse prognostic factor for several tumours [15–17]. Furthermore, a relevant hallmark of cancer is the epithelial-to-mesenchymal transition (EMT), which describes the process of cancer cells to lose their cell polarity and adhesion and acquires invasive and migratory properties [18]. Several proteins play key roles in this process, comprising E-cadherine, β-catenin and vimentin. Vimentin is expressed only in mesenchymal cells and may affect DNA transcription and cell apoptosis [18].

It would be crucial for modern patient care if imaging can reflect these distinctive tumor features to better characterize tumor biology in a non-invasive manner. Yet, it is unclear, whether histogram parameters derived from DCE-MRI are associated with the amount of TIL, tumor-stroma ratio and vimentin expression in HNSCC. Therefore, the present study sought to elucidate possible associations between histogram parameters derived from DCE-MRI and stroma to parenchyma ratio, EMT and TIL in HNSCC.

## Materials and methods

This study is a retrospective analysis of a prospectively acquired patient sample of HNSCC patients. It was approved by the institutional review board (Ethic committee of the University of Leipzig, study codes 180–2007, 201–10–12,072,010, and 341–15–05102015) and every patient gave their written consent.

### Patients

Overall, 26 patients with primary HNSCC of different localizations were involved in the study. There were 7 (28.6%) women and 19 (71.4%) men with a mean age of 56.7 ± 10.2 years, range 33–77 years. The patients received no specific treatment before the MRI and the biopsy. Table 1 gives an overview of the included patients.

**Table 1:** Overview of the patient sample

Localization	n (%)
Oropharynx	11 (42.3)
Tonsil	7 (26.9)
Hypopharynx	4 (15.4)
Larynx	4 (15.4)
Grading	
Moderate (G2)	11 (42.3)
Poor (G3)	15 (57.7)
Stage	
T2	7 (26.9)
T3	10 (38.5)
T4	9 (34.6)
Nodal status	
Nodal positive	23 (88.5)
Nodal negative	3 (11.5)
HPV status	
HPV positive	15 (57.7)
HPV negative	11 (42.3)

### DCE-MRI

DCE-MRI was obtained with a 3 T scanner (Siemens Biograph mMR; Siemens Healthcare, Erlangen, Germany) within a clinical MRI protocol. In every case, dynamic T1-weighted DCE sequence (TR/TE 2.47/0.97 ms, slice thickness 5 mm, flip angle 8°, voxel size 1.2 × 1.0 × 5.0 mm) included 40 subsequent scans every 6 s were applicated. Intravenous administration of contrast medium (Gadovist®, Bayer Healthcare, Leverkusen, Germany) in a dose of 0.1 mmol per kg of body weight was started after the fifth scan at a rate of 3 ml per second and flushing with 10 ml of normal saline using a power injector (Spectris Solaris, Medrad, Bayer Healthcare, Leverkusen, Germany). After this, all acquired images were transferred to a software module for tissue perfusion estimation (Tissue 4D, Siemens Medical Systems, Erlangen, Germany). The software offers a population-based approach for the arterial input function (AIF) and the best of three available AIF-options was chosen according to the result of the chi2-parameter, which serves as an error measure for the model fit [9].

**Fig. 1:**
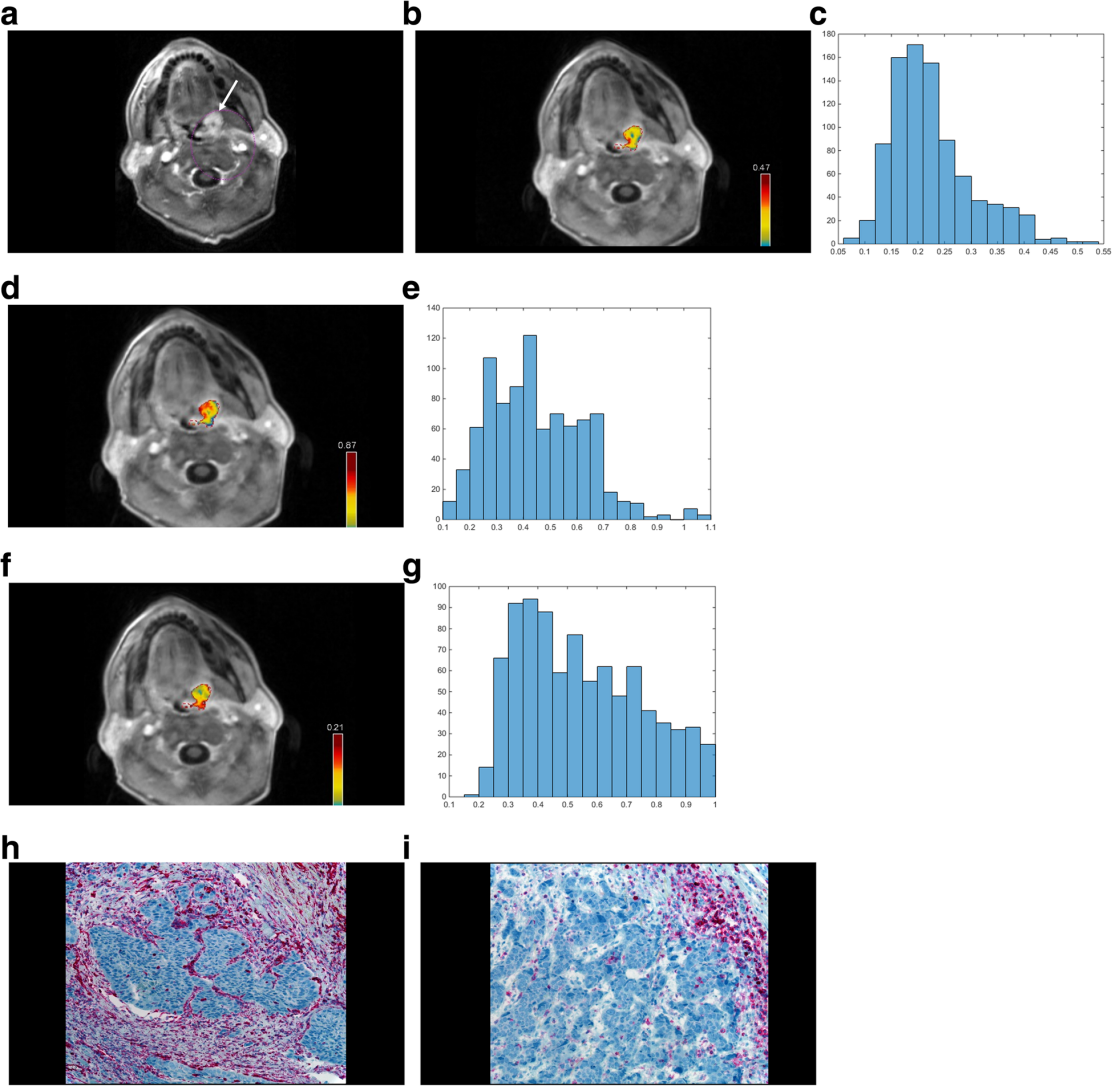
Representative case of the patient sample with a tumor of the left tonsil. A. T1-weighted sequence after contrast media application with fat saturation. **A.** tumor of the left tonsil with relative homogeneous contrast enhancement can be appreciated. **B.** Color coded map of the K^trans^ values. An inhomogenous tumor can be appreciated with over all intermediate K^trans^ values. The tumor is measured on all tumor displaying slices. C. The corresponding histogram of the K^trans^ values (all parameters are given in 1/min) with the resulting values: mean 0.22, minimum 0.06, maximum 0.5, P10 0.14, P25 0.17, P75 0.26, P90 0.34, median 0.20, mode 0.20, kurtosis 3.93, skewness 1.01, entropy 3.23. D. Color coded map of the Kep values. Overall, homogenously intermediate to high Kep values can be seen. E. The corresponding histogram of the Kep values (all parameters are given in 1/min): mean 0.45, minimum 0.1, maximum 1.09, P10 0.23, P25 0.31, P75 0.57, P90 0.68, median 0.42, mode 0.29, kurtosis 3.23, skewness 0.60, entropy 3.72. F. Color coded map of the Ve values. Inhomogenously, intermediate Ve values can be appreciated. G. The corresponding histogram of the Ve values (unitless). Mean 0.55, minimum 0.19, maximum 1.0, P10 0.31, P25 0.38, P75 0.71, P90 0.85, median 0.52, mode 0.3, kurtosis 2.13, skewness 0.42, entropy 3.87. H. The corresponding vimentin-stained specimen (20-fold magnification) of this patient. There is no relevant staining of this specimen. The stroma/tumor ratio is 40:60. I. The corresponding CD45-stained specimen. The expression of TIL in the tumor compartment is 1% and within the stroma compartment is 50%

The following parameters were calculated: - K^trans^ or volume transfer constant representing the diffusion of contrast medium from the plasma through the vessel wall into the interstitial space; − V_e_ or volume of the extravascular extracellular space (EES);- K_ep_ or parameter for diffusion of contrast medium from the EES back to the plasma. On the next step, the saved DICOM images were processed using a custom-made Matlab-based application (The Mathworks, Natick, MA, USA). Then, polygonal regions of interest were semi-automatically drawn along the contours of the primary tumor on every slide with tumor (whole lesion measurement) in accordance with the tumor boundary defined by the T1-weighted images after contrast enhancement. All measures were performed by one experienced author (A.S., 18 years of general radiological experience) blinded to the histopathology results. The following histogram parameters were calculated for K^trans^, K_ep_ and V_e_, respectively: mean, maximum, minimum, median, mode, 10th, 25th, 75th and 90th percentile as well as kurtosis, skewness, and entropy. These procedures were described previously [19]. Figure 1 displays a representative case of the present study. A second reader with 5 years of general experience in radiology (H.J.M.) performed an independent calculation of the histogram parameters for all tumors to perform an interreader agreement.

### Histopathological analysis

The biopsies were obtained of the primary tumor before any form of treatment. The histopathology analysis was performed by an experienced board-certified pathologist (A.K.H.) blinded to the imaging results. The biopsy specimens were deparaffinized, rehydrated and cut into 5 μm slices. First, the standard Hematoxylin and eosin (HE) stained was evaluated. Thereafter, the histological slices were stained by vimentin (DAKO, clone Vim 3B4, dilution 1:200), and Leucocyte common antigen (LCA) (CD45, DAKO, dilution 1:150). All calculations were made on a 20-fold magnification. Tumor-stroma ratio was evaluated on the HE stained specimen and percentages of the high-power field were given per tumor and stroma part. Stroma-rich and tumor-rich tumors were grouped accordingly to a threshold-value of 50% in accordance with previous studies [20, 21].

For the TIL expression, the LCA stained specimen were used. The positive stained areas were evaluated for the stroma and tumor area independently. Next, for vimentin expression the percentage of stained area was calculated. Lastly, *Human papillomavirus* (HPV) status was evaluated with p16 expression (p16 expression, CINtec Histology, Roche, Germany).

### Statistical analysis

Statistical analysis was performed using performed using GraphPad Prism 5 (GraphPad Software, La Jolla, CA, USA). Collected data were evaluated by means of descriptive statistics. Mean values were stated with standard deviation in all instances.

Spearman’s correlation coefficient (r) was used to analyze associations between investigated parameters. A two-sided Mann-Whitney-Test was used to test between groups. Intraclass coefficients (ICC) were used to test for interreader variability. *P*-values < 0.05 were taken to indicate statistical significance.

## Results

Table 2 summarizes the investigated histogram parameters derived from DCE-MRI and provides the interreader variability.

**Table 2:** Overview of the investigated histogram parameters derived from Dynamic-contrast enhanced MRI

Parameters	Mean ± Standard Deviation	Range	Intraclass coefficient (95% Confidence interval)
**Ktrans (1/min)**			
Mean	0.19 ± 0.11	0.06–0.53	0.97 (0.94–0.97)
Min	0.05 ± 0.03	0.009–0.13	0.97 (0.93–0.98)
Max	0.63 ± 0.52	0.10–2.92	0.98 (0.95–0.99)
P10	0.10 ± 0.06	0.04–0.24	0.97 (0.93–0.99)
P25	0.13 ± 0.08	0.05–0.35	0.90 (0.79–0.96)
P75	0.23 ± 0.13	0.08–0.63	0.98 (0.95–0.99)
P90	0.30 ± 0.17	0.08–0.91	0.66 (0.35–0.83)
Median	0.18 ± 0.10	0.07–0.45	0.96 (0.95–0.99)
Mode	0.15 ± 0.10	0.04–0.47	0.93 (0.91–0.98)
Kurtosis	6.43 ± 6.73	2.70–33.70	0.98 (0.97–0.99)
Skewness	1.12 ± 0.83	−0.25-3.85	0.97 (0.93–0.98)
Entropy	3.33 ± 0.51	2.10–4.72	0.97 (0.94–0.98)
**Kep (1/min)**			
Mean	0.40 ± 0.17	0.15–0.89	0.93 (0.85–0.97)
Min	0.09 ± 0.06	0.01–0.22	0.95 (0.88–0.98)
Max	1.04 ± 0.56	0.31–2.97	0.92 (0.84–0.96)
P10	0.22 ± 0.11	0.07–0.58	0.95 (0.88–0.98)
P25	0.29 ± 0.13	0.10–0.72	0.98 (0.95–0.99)
P75	0.49 ± 0.20	0.19–1.04	0.95 (0.88–0.98)
P90	0.59 ± 0.23	0.23–1.19	0.97 (0.94–0.98)
Median	0.38 ± 0.16	0.15–0.89	0.96 (0.92–0.98)
Mode	0.35 ± 0.16	0.14–0.89	0.96 (0.91–0.98)
Kurtosis	4.61 ± 4.91	2.20–26.81	0.98 (0.97–0.99)
Skewness	0.63 ± 0.61	−0.04-2.52	0.97 (0.92–0.98)
Entropy	3.43 ± 0.56	2.20–4.38	0.96 (0.90–0.98)
**Ve (unitless)**			
Mean	0.52 ± 0.18	0.16–0.78	0.98 (0.95–0.99)
Min	0.16 ± 0.10	0.03–0.34	0.75 (0.50–0.88)
Max	0.94 ± 0.15	0.44–1.00	0.91 (0.81–0.96)
P10	0.31 ± 0.15	0.09–0.58	0.92 (0.83–0.96)
P25	0.38 ± 0.17	0.12–0.70	0.96 (0.94–0.98)
P75	0.64 ± 0.21	0.19–0.92	0.92 (0.94–0.98)
P90	0.77 ± 0.21	0.25–0.97	0.95 (0.88–0.98)
Median	0.49 ± 0.20	0.15–0.83	0.98 (0.95–0.99)
Mode	0.42 ± 0.24	0.12–0.98	0.98 (0.96–0.99)
Kurtosis	3.04 ± 1.14	1.89–5.37	0.95 (0.89–0.98)
Skewness	0.42 ± 0.70	−1.41-1.55	0.95 (0.89–0.98)
Entropy	3.29 ± 0.63	1.55–4.12	0.93 (0.85–0.97)

Abbreviation *P* percentile

The mean value of stroma-ratio was 26.5 ± 16.0%, and the mean value of tumor-ratio was correspondingly 73.5 ± 16.0%. The mean value of vimentin expression was 29.9 ± 36.6%. The mean TIL expression of the stroma compartment was 34.2 ± 31.2% and it was 6.4 ± 13.7% of the tumor compartment. The TIL expression of the stroma compartment was significantly higher compared to the tumor compartment (*p* = 0.0006).

**Fig. 2:**
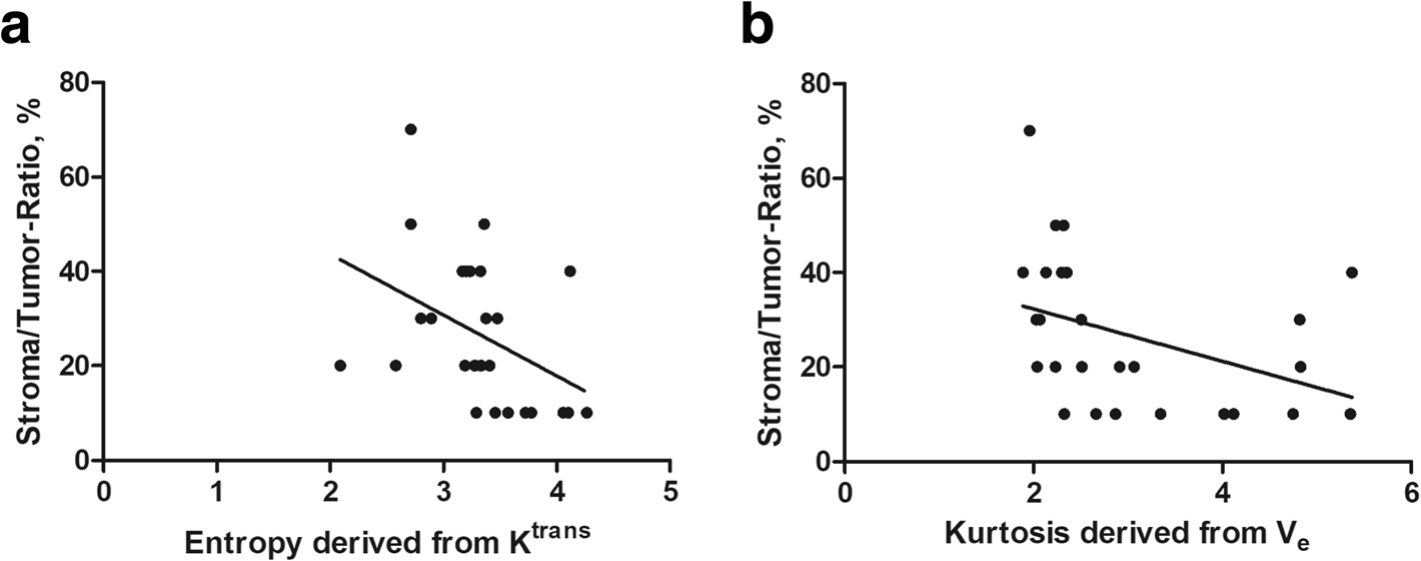
**a**. Spearman’s correlation analysis between the stroma/tumor ratio and the entropy derived from K^trans^ (*r* = − 0.52, *p* = 0.0071). **b.** as well as for kurtosis derived from Ve (*r* = − 0.53, *p* = 0.0058)

Tumor-stroma ratio correlated with entropy derived from K^trans^ (*r* = − 0.52, *p* = 0.0071) and kurtosis derived from V_e_ (*r* = − 0.53, *p* = 0.0058) (Fig. 2). No statistically significant correlations were identified for other investigated parameters.

Vimentin expression did not significantly correlate with DCE-MRI parameters.

**Fig. 3:**
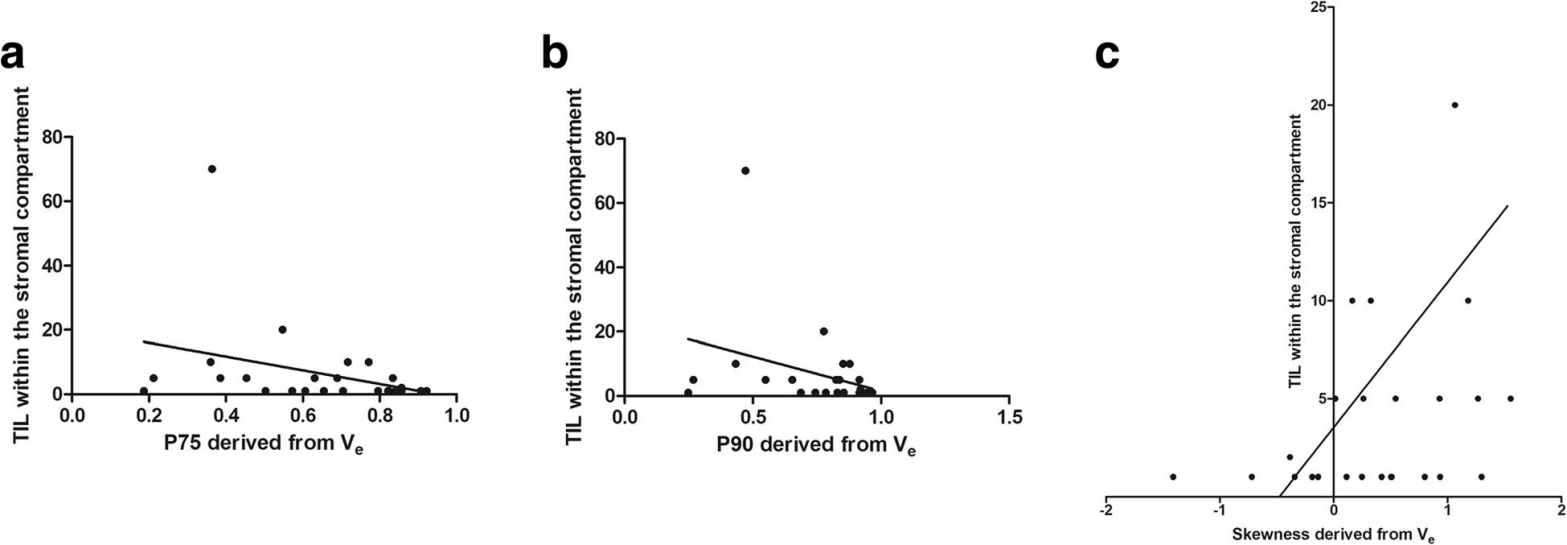
Spearman’s correlation analysis between the expression of TIL within the stroma compartment and different V_e_ values. **a.** p75 derived from Ve (*r* = − 0.40, *p* = 0.046), **b.** p90 derived from Ve (*r* = − 0.44, *p* = 0.03) **c.** and for skewness (*r* = 0.43, *p* = 0.03)

Several V_e_ derived parameters correlated well with expression of TIL within the stroma compartment: p75 (*r* = − 0.40, *p* = 0.046), p90 (*r* = − 0.44, *p* = 0.03), and skewness (*r* = 0.43, *p* = 0.03) (Fig. 3). No K^trans^ or K_ep_ parameters showed significant correlations with TIL within the stroma compartment.

**Fig. 4:**
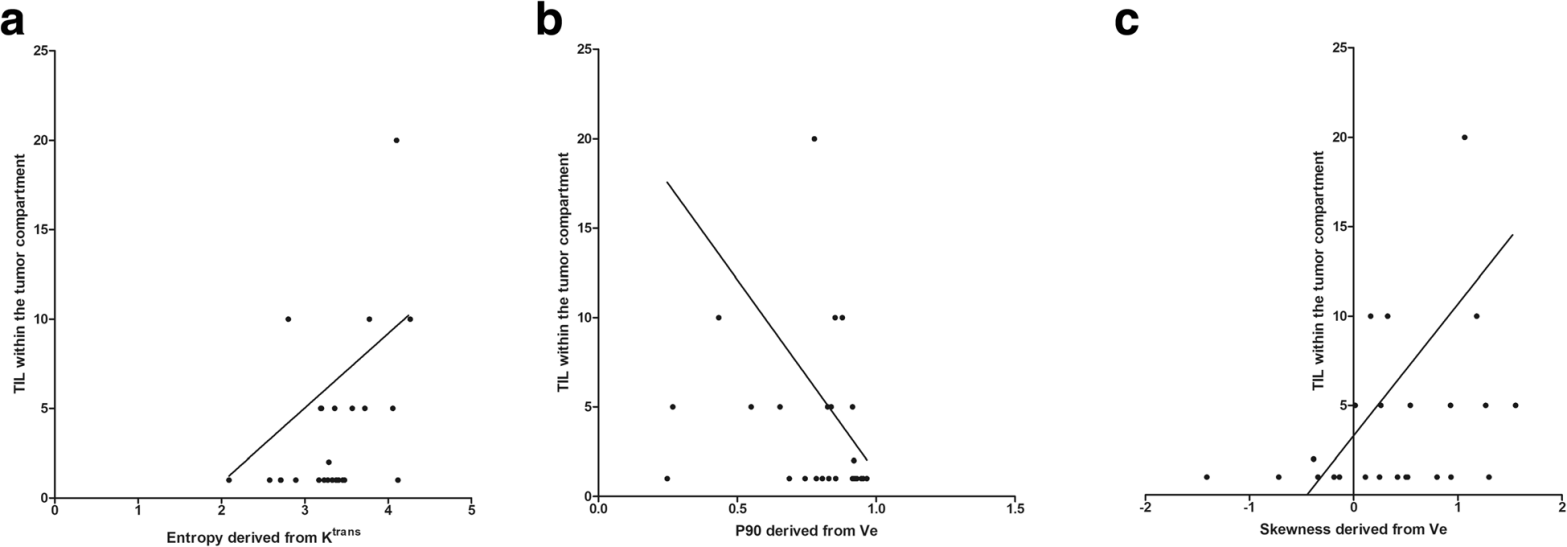
Spearman’s correlation analysis between the expression of TIL within the tumor compartment and several DCE-MRI values: a Entropy derived from K^trans^ (*r* = 0.39, *p* = 0.047), b p90 derived from Ve (*r* = 0.41, *p* = 0.036). c Skewness derived from Ve (*r* = 0.41, *p* = 0.037)

For expression of TIL within the tumor compartment, there were several associations as follows: entropy derived from K^trans^ (*r* = 0.39, *p* = 0.047), p90 derived from V_e_ (*r* = 0.41, *p* = 0.036) and skewness derived from V_e_ (*r* = 0.41, *p* = 0.037) (Fig. 4).

There were no significant differences of DCE-MRI values between tumor groups of high and low tumor-stroma ratio using the threshold value of 50%.

### Subanalysis according to HPV status

**Fig. 5:**
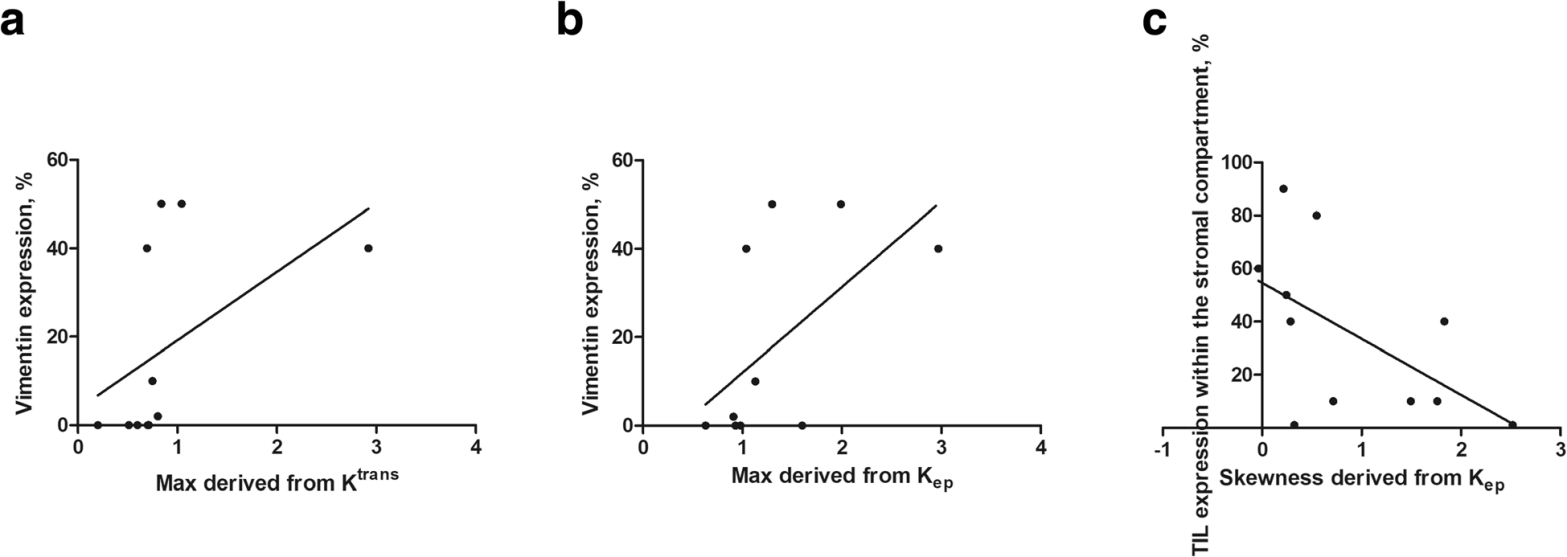
Subanalysis of HPV negative tumors. DCE-MRI values: a Spearman’s correlation analysis between vimentin expression and K^trans^ max (*r* = 0.76, *p* = 0.007). b Spearman’s correlation analysis between vimentin expression and Kep max (*r* = 0.65, *p* = 0.03). c Spearman’s correlation analysis between Skewness derived from K_ep_ and TIL expression within the stromal compartment (*r* = − 0.65, *p* = 0.03)

In the HPV negative tumor group (*n* = 11), vimentin expression correlated strongly with K^trans^ max (*r* = 0.76, *p* = 0.007) as well as with K_ep_ max (*r* = 0.65, *p* = 0.03) (Fig. 5). Furthermore, skewness derived from K_ep_ correlated with the TIL expression within the stromal compartment (*r* = − 0.65, *p* = 0.03) (Fig. 5c). No DCE-MRI parameters correlated with TIL expression within the tumoral compartment and the tumor-stroma ratio.

In the HPV positive tumor group (*n* = 15), there were no statistically significant associations with vimentin expression and TIL expression within the stromal compartment.

**Fig. 6:**
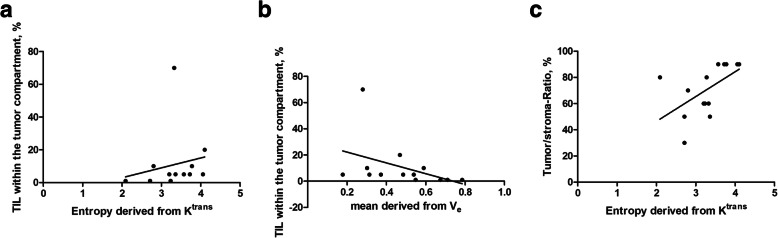
Subanalysis of HPV negative tumors. DCE-MRI values: a Spearman’s correlation analysis between TIL expression within the tumor compartment and entropy derived from K^trans^ (*r* = 0.61, *p* = 0.03). b Spearman’s correlation analysis between TIL expression within the tumor compartment and mean derived from V_e_ (*r* = − 0.66, *p* = 0.01). c Spearman’s correlation analysis between tumor-stroma ratio and entropy derived from K^trans^ (*r* = 0.67, *p* = 0.009)

TIL expression within the tumoral compartment correlated well with entropy derived from K^trans^ (*r* = 0.61, *p* = 0.03) (Fig. 6a). Furthermore, several parameters derived from V_e_ also correlated statistically significant with_._ TIL expression within the tumoral compartment, the strongest correlation coefficient was shown for V_e_ mean (*r* = − 0.66, *p* = 0.01) (Fig. 6b).

Additionally, entropy derived from K^trans^ correlated well with the tumor-stroma ratio (*r* = 0.67, *p* = 0.009) (Fig. 6c). Moreover, several parameters derived from V_e_ also correlated with tumor-stroma ratio. The strongest correlations were observed for V_e_ kurtosis (*r* = 0.59, *p* = 0.02) and V_e_ p90 (*r* = − 0.64, *p* = 0.01).

## Discussion

The present study identified interesting associations between DCE-MRI and histopathology in HNSCC. These findings give a deeper understanding of the prediction of histopathology features by functional imaging techniques. To clarify, DCE-MRI is a functional imaging modality, which measures the sequential changes of signal intensity over time after contrast media application using a T1-weighted sequence [4, 6, 7]. According to the literature, the derived parameters of DCE-MRI can reflect different aspects of the tumor biology [9]. Beyond that, these parameters can further be analyzed by the histogram analysis technique, which can quantify the heterogeneities of tumors. There is enough evidence that histogram parameters derived from DCE-MRI are capable to reflect tumor microstructure, especially vessel density in tumor tissues, which was also shown for HNSCC [5, 8, 12]. Of note, the different DCE-MRI parameters reflect different histopathologic features of tumors. For example, as reported previously, K_ep_ correlated stronger with vessel density in HNSCC compared to the other DCE-MRI parameters [12]. Moreover, parameters derived from K^trans^ and K_ep_ can reflect proliferation potential [8].

Interestingly, even neoangiogenesis related immunohistochemical parameters, such as vascular endothelial growth factor and hypoxia inducible factor 1-alpha showed associations with histogram parameters in HNSCC [11, 13]. In a recent study, investigating several immunohistochemical features, only a correlation between K^trans^ and p53 was identified [7]. Importantly, K^trans^ has been validated as an independent prognostic factor for overall survival in patients with HNSCC undergoing radio-chemotherapy [22].

The fact that especially V_e_ related parameters were associated with TIL of tumor and stroma compartments might be explained by the ability of V_e_ parameters to reflect cellularity in tumors [10, 23–25]. For K^trans^, entropy was significantly associated with TIL.

Entropy is a parameter, which quantifies the heterogeneity of the histogram and is often discussed to also reflect the heterogeneity of the tumors. Recently, a significant correlation between K^trans^ and epidermal growth receptor expression was reported in p16 positive HNSCC (*r* = 0.50, *p* = 0.03) [26].

The present study showed that DCE-MRI can also predict tumor-stroma ratio with entropy derived from K^trans^ and V_e_ derived parameters. This finding is of clinical importance. In fact, for oral tongue cancers, a low tumor-stroma ratio was shown to have negative prognostic value in terms of disease-specific survival, with a hazard ratio of 1.9 (*p* = 0.039), and overall survival (hazard ratio of 1.7, *p* = 0.044), independently of other investigated parameters [20].

An important novel aspect of the present study is that the associations between DCE-MRI derived parameters with vimentin and tumor-stroma ratio differ significantly between HPV negative and positive HNSCC. In a previous study it could be shown that histogram analysis parameters derived from diffusion-weighted imaging were different between HPV positive and negative cancers [26]. This was explained by different microstructure of the tumors according to the HPV status [26].

To date, there are few studies investigating possible association between imaging parameters and TIL in oncology [27–29]. Presumably, imaging can reflect TIL expression when different imaging modalities are used together and even supported by artificial intelligence. More studies are needed to confirm this hypothesis.

In previous studies the complex interactions between histogram parameters derived from MRI and TIL expression and tumor-stroma ratio was investigated [28, 29]. For diffusion-weighted imaging, kurtosis and skewness were significantly associated with tumor-stroma ratio, whereas entropy correlated significantly with the TIL expression of the tumor compartment [28]. Another study tried to quantify morphological T1- and T2- images employing the histogram approach and could elucidate several significant associations [29]. In this mentioned study, T1- derived correlated with TIL expression within the stromal compartment [29]. One hypothesis was, whether quantified T1-signal intensity curves in the DCE-MRI method can better reflect histological features in HNSCC. Interestingly, the identified correlation coefficients between several DCE-MRI related values and TIL expression within the stromal compartment in the present study are slightly better than the abovementioned study. This is a key aspect of the present study that DCE-MRI can better reflect tumor features than the histogram approach derived from morphological images.

To summarize, it is unclear how precise the prediction of tumor features can become with MRI. Moreover, complex analysis techniques with radiomics and a multiparametric approach might harbor better results, which then might result in a clinical translation.

Another point is also to consider. HNSCC is a highly heterogeneous tumor with different biological behavior comprising different localizations with genetically defined subtypes [30]. Clearly, more, and larger studies are needed to define the correlations between imaging and histopathology.

An important aspect for the translation of imaging biomarkers into clinical routine is the interreader variability. The present results could show that histogram parameters derived from DCE-MRI can be considered robust with a good to excellent interreader agreement. This is in good agreement with previous studies regarding histogram parameters in HNSCC [29, 31, 32].

There are several limitations of the present study to address. First, it is a retrospective analysis performed on a prospectively recruited patient sample. However, the imaging and pathology analyses were performed blinded and independently to each other to reduce possible bias. Second, the imaging analysis was performed as a whole tumor measurement, whereas the histopathology analysis was performed on bioptic specimens. This might result in certain spatial incongruencies, which yet represents daily clinical practice.

## Conclusion

DCE-MRI might be able to reflect tumor compartments and TIL expression in HNSCC. The most promising parameters were K^trans^ and V_e_ related values.

## Data Availability

The datasets used and/or analyzed during the current study are available from the corresponding author on reasonable request.

## Abbreviations

HNSCC: Head and neck squamous cell cancer
ADC: Apparent Diffusion coefficient
DWI: Diffusion weighted imaging
DCE-MRI: Dynamic contrast enhanced magnetic resonance imaging
